# Data on Swiss fruit and wine growers’ management strategies against *D. suzukii*, risk preference and perception

**DOI:** 10.1016/j.dib.2019.103920

**Published:** 2019-04-17

**Authors:** Ladina Knapp, Esther Bravin, Robert Finger

**Affiliations:** a Agricultural Economics and Policy Group, ETH Zurich, Zurich Switzerland; b Competence Division for Research Technology and Knowledge Exchange Plants and Plant Products, Agroscope, Wädenswil, Switzerland

**Keywords:** Risk management strategies, *D. suzukii*, Risk preferences, Locus of control, Self-efficacy, Grapes, Plums, Berries, Cherries, Switzerland

## Abstract

The survey data presented in this article provides information on risk management strategies in response to the invasive pest *Drosophila suzukii (spotted wing drosophila)* collected among Swiss fruit and wine growers. The survey covered grape, plum, berry and cherry growers and the years 2016, 2017 and 2018. Strategies to prevent or control *Drosophila suzukii* were collected at the variety level and information on perceived infestation levels as well as harvest losses was collected. In total, nine surveys were conducted, creating a unique panel dataset. Additionally, data was collected with regard to fruit growers’ characteristics (e.g. age, gender) and farm characteristics (e.g. farm size, farm-level workforce, succession, insurance use). Risk preferences were elicited via a self-assessment of risk preferences and (for selected surveys) using a multiple price list. Additionally, (for selected surveys) locus of control and self-efficacy were evaluated via self-assessment questions.

**Table:** Specifications table

Subject area	Agricultural Risk management
More specific subject area	Risk management strategies against *D. suzukii*, risk preferences, locus of control, self-efficacy
Type of data	CSV file
How data was acquired	Online survey
Data format	Raw data
Experimental factors	Fruit growers growing the specific fruit (e.g. grapes, plums, berries and cherries)
Experimental features	The data was collected in an online survey among fruit growers in Switzerland.
Data source location	Switzerland
Data accessibility	Data is accessible via ETH Research Collection: http://hdl.handle.net/20.500.11850/292794
Related research article	L. Knapp, D. Mazzi, R. Finger, Management strategies against *Drosophila suzukii*: Insights into Swiss grape growers' choices. Pest. Manag. Sci., https://doi.org/10.1002/ps.5397.

**Table:** 

**Value of the data** • The data highlights which measures were taken against *D. suzukii* and the perception of infestation level by *D. suzukii* as well as harvest losses for grapes, plums, berries and cherries. • The data enables to understand a wide range of risk management strategies undertaken by fruit and wine growers and associate it to their risk preferences as well as farm and fruit growers' characteristics. • The data provides insights in growers risk preferences and thus allows comparison with other studies. Likert scale self-assessment questions and multiple price lists are used. Risk perception was also measured. • The data also allows to link risk perception and preferences to self-efficacy and locus of control.

## 1 Data

We collected data on risk management strategies undertaken by fruit growers against the *Drosophila suzukii,* an invasive vinegar fly mainly infesting thin-skinned fruits [1].

We present nine datasets as seen in Table 1, resulting from nine online surveys undertaken with fruit growers in Switzerland from 2016 to 2018. Based on these nine surveys, we present unbalanced panel datasets for grapes, cherries and plums. The surveys, datasets and the codebooks describing the variables are available online on the ETH Zürich Research Collection: http://hdl.handle.net/20.500.11850/292794.

**Table 1:** Overview of the number of participants per fruit surveys and year.

Fruits	2016	2017	2018
Grapes	372	331	389
Plums	112	74	91
Berries	not conducted	50	not conducted
Cherries	not conducted	94	109

Notes: Berries and cherries were not included in 2016 given administrative reasons of the project and it was not undertaken for berries in 2018 given the small amount of participants in 2017.

**Fig. 1:**
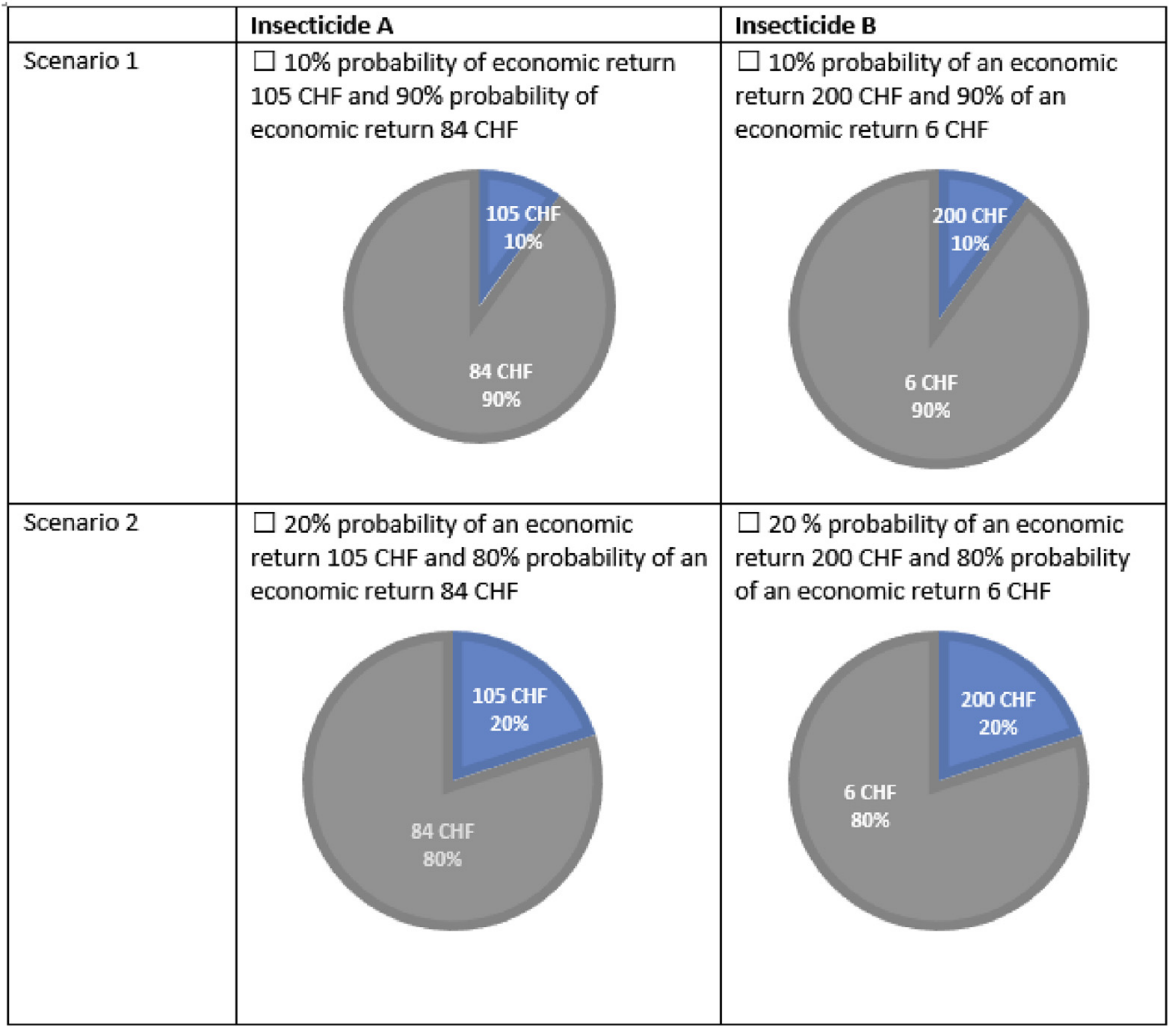
Example of the multiple price list.

In the surveys, a wide range of strategies to prevent or control *Drosophila suzukii* was collected at the variety level. Moreover, information on their perceived infestation levels as well as harvest losses due to *Drosophila suzukii* were obtained. A wide range of other farm (e.g. size, production system, tenure) and fruit growers’ characteristics (e.g. age, gender) was collected. In all three years from 2016 to 2018, risk preferences were elicited using contextualized self-assessment questions on attitude to risk taking in four different domains (production, market and prices, external financing and agriculture in general) [2]. A simple 11 Likert scale assessment question was used following [3]. For data collected in 2018, also a multiple price list following [4] was used (see Fig. 1). Furthermore, the 2018 data includes information on locus of control and self-efficacy of fruit growers following [5], [6].

## 2 Experimental design, materials, and methods

Nine online surveys available in German, French and Italian were distributed via a link sent to the fruit and wine growers by email in collaboration with a number of Swiss cantonal agricultural services from 2016 to 2018. The month the surveys were sent out usually varied (Mid October for berries; Beginning of November for cherries and plums; Mid November for grapes). The timing of surveys was connected to harvest dates, which varied across fruits and years. All surveys were sent out once the fruits were already harvested for that given season. The link was also sent by the periodic plant protection recommendation issued by the Swiss Centre of excellence for agricultural research (Agroscope). For stoned fruit and berries it was additionally sent out in the Newsletter of the Swiss Fruit Union. We used the online platform LIMESURVEY^1^ to design our survey. Each survey was pre-tested before being sent out for the first time with fifteen experts from cantonal advisory services, the Swiss Task Force Drosophila suzukii^2^ and fruit growers (either grape, plum, berry or cherry growers depending on the survey). Surveys were online for one month, although in some cases the period was prolonged to one month and a half in order to increase participation rates. As an incentive for all years (2016–2018), we provided individual feedback on the survey's results to every fruit grower who was interested following [7]. The individual feedback consisted of aggregated information on the whole sample on management strategies undertaken against the *D. suzukii* in relation to their individual response to the question. As a further incentive, for years 2016–2017, we indicated that four purchase vouchers of a value of 50 Swiss Francs (CHF) could be won by fruit growers. For the 2018 surveys, we had an incentive based on real payouts from the lottery (multiple price list, see below).

The surveys questions varied with a minimum of 37 questions and a maximum of 51 questions, depending on crop and year. In 2018 for instance some technical questions were deleted and other questions were added (e.g. self-assessment on locus of control and self-efficacy/multiple price list). The vast majority of questions was, however, included in all three years. On average, fruit growers needed around 30 minutes to complete the surveys. The surveys were structured in following sections:

i. Varieties of fruits, cultivated areas
ii. Perceived infestation levels of *D. suzukii*
iii. Measures taken against the *D. suzukii*
iv. Farm characteristics
v. Fruit growers' characteristics

### 2.1 Varieties of fruits, cultivated areas

The surveys included questions on the varieties of fruits and the cultivated area for each variety. Variety specific questions reflect the large heterogeneity of the susceptibility of the varieties to *D. suzukii* fly. The number of varieties included in the survey was determined by the most important varieties used in Switzerland, ranging from minimum 8 varieties (for berries) to maximum 30 varieties (for grapes).

### 2.2 Perceived infestation levels of D. suzukii

Fruit growers were asked per variety to assess the *D. suzukii* infestation level. The intervals chosen for the infestation levels were based on expert interviews for the given fruit.

### 2.3 Measures taken against the D. suzukii

Fruit growers were asked which measures they undertook to prevent or control *D. suzukii* infestation*.* Measures were the same for all fruits, namely sanitation measures, control of infestation, nets, mass trapping of flies, insecticides, early harvest, none of these strategies and other strategies. For berries, two measures were however different, namely short interval harvest instead of early harvest and lime application which was an additional option. We also asked more specific questions for every measure, in order to gain more detail and technical knowledge on the precise measures used (e.g. the type of insecticides), however these were different for all fruits. We further asked fruit growers how much they estimate their additional costs in percentage per kg yield of fruit due to the measures taken against the *D. suzukii*. Questions were also asked on the satisfaction of the fruit growers with their strategies against the *D. suzukii* and which measures they will continue undertaking in the future. In this section, we also included a question on the sources of information fruit growers rely on for *D. suzukii* expertise (e.g. Internet, Agroscope, cantonal offices, other fruit growers).

In the surveys, we included a question on the perception of risk originating from *D. suzukii* for the next year harvest, by asking fruit growers to indicate the percentage of yield loss and additional expected costs due to measures undertaken against the *D. suzukii* for the coming year. These percentage intervals were changed for years 2017 and 2018 from a 25% interval to a 5% interval to get more variance in the answers received from fruit growers.

### 2.4 Farm characteristics

Farm characteristics were also included in the surveys such as the location (using the postal code) of the farm, the specialization of the farm (mixed or specialized), the production system (organic/conventional), the farms ‘total work force availability, the total farmland, the share of farmland rented out and use of hail and/or frost insurance. For all surveys, we added a question on how the product is marketed. These marketing options were different according to the fruit and the marketing options available in Switzerland (e.g. direct marketing, large distributor). We asked which other more general risk-management strategies the fruit grower is following on the farm, for instance whether fruit growers are undertaking diversification activities or off farm investments. In order to understand the importance of the fruit production for the fruit grower, we included a question on the percentage of earning coming from farming as well as how much the production of the given fruit contributes to the fruit growers’ earning in percentage.

### 2.5 Fruit growers’ characteristics

We included questions related to the year of birth, gender and whether the fruit grower already has a planned successor for the farm. In the last section of the surveys, risk preferences were measured in two different ways. For the years 2016–2017, risk preferences were measured via Likert type contextualized self-assessment questions on attitude to risk taking in four different domains, namely production, market and prices, external financing and agriculture in general, following [8]. Participants were asked to choose a value from 0= not willing to take a risk at all to 10=very willing to take a risk in the chosen domain following [3].

For the surveys 2017–2018, some technical questions were deleted whereas new questions were added in order to adapt to the actual situation, for instance whether fruit growers faced frost damage on their harvest and whether they undertook the option of insurance against hail and frost.

For the year 2018, we added a section on risk preferences measurement with a contextualized multiple price list following [4]. The wording of the questions was adapted to the farm contextualization and the *D. suzukii* infestation, namely:

“You can decide between two insecticides against the *Drosophila suzukii*, A and B. Consider that the overall costs, the payment time, the difficulty in handling the insecticide, the safety of the insecticide for the consumers and producers are the same for insecticide A and insecticide B.

Insecticide A has a more stable economic return than B, given that it has been longer on the market and we can better predict the probability of the economic return. Insecticide B is new on the market; it has a less predictable economic return but reveals itself to be in some cases extremely efficient and thus provides you at times higher economic returns. Below in the table, there are 10 different scenarios. You are asked to choose either A or B. Note that no choice is right or wrong and all depends on your personal preferences.”

We followed Meraner and Finger (2017) concerning the illustration of the lottery and the design of the lottery [8]. Participants were instructed that 10% of the participants will be selected as winners. From the ten scenarios, we informed participants that one scenario would be randomly drawn for each of the winners and the scenario will be played out based on the choice of the insecticide A or B. Winners of the lotteries were then contacted via email and we asked for their bank account information to transfer the amount won. As can be seen in Fig. 1, the minimum that participants could win was 6 CHF and the maximum was 200 CHF. The expected return for each participating fruit grower was 15 CHF.

Finally, for the survey 2018 we added questions on locus of control [9] and self-efficacy [6] as these concepts may contribute explaining fruit growers’ (risk) management decisions. Locus of control and self-efficacy were measured via Likert type questions all related to the agricultural production sector and the fruit in question. We built our questions on locus of control following [5] and self-efficacy following [6]. Overall, we had three questions on locus of control and four questions on self-efficacy where fruit growers could select from 0 = I do not agree to 10 = I fully agree.

There were only two open questions in the surveys, one regarding comments and another asking whether they have found any special findings with regard to the *D. suzukii.*

To ensure anonymity of the participants, we deleted the postal code and kept the information at the cantonal level in Switzerland. Additionally, we also deleted the email addresses from the dataset and written comments.

## Footnotes

1 www.limesurvey.org.

2 www.drosophilasuzukii.agroscope.ch.
